# Comprehensive geriatric assessment and palliative care

**DOI:** 10.1002/agm2.12367

**Published:** 2024-10-16

**Authors:** Alberto Castagna, Vincenzo Militano, Carmen Ruberto, Ciro Manzo, Giovanni Ruotolo

**Affiliations:** ^1^ ASP di Catanzaro, Primary Care Department Catanzaro Italy; ^2^ AOU “Renato Dulbecco,” SOC di Medicina Nucleare Catanzaro Italy; ^3^ AOU “Renato Dulbecco,” SOC di Geriatria Catanzaro Italy; ^4^ ASL Napoli 3, Internal and Geriatric Department, HD n 51 Pomigliano d'Arco Italy

**Keywords:** geriatric palliative care, guidelines, palliative care comprehensive geriatric assessment

## Abstract

The geriatric vision of palliative care is based on a multidisciplinary, patient‐centered approach, looking for a balance between human dignity and medical treatments with a particular focus on the social and ethical aspects. In order to develop the best care models, there is a rising need for a tighter collaboration of all the involved players (i.e., doctors, nurses, social workers). Indeed, the idea of a fragmented system without considering the patient or his/her family is not at all applicable to older patients with chronic disease. The causes of death, the phase of death changes, and the extend of last period of life could be a long phase characterized by complicated treatment decisions, difficult management of symptoms, multiple psychosocial problems, and complex spiritual distress. Recently, Italian guidelines on Comprehensive Geriatric Assessment (CGA) have been published. However, none of the identified studies on patients in hospice and other palliative care facilities met the criteria for inclusion. These findings underscore the need for further research to determine the potential benefits of a multidimensional approach for patients in hospice and other palliative care settings. Our reflections and suggestions on the CGA use for older persons in palliative care may be a starting point for an open and continuous dialogue with all the operators concerned.

## Graphical abstract

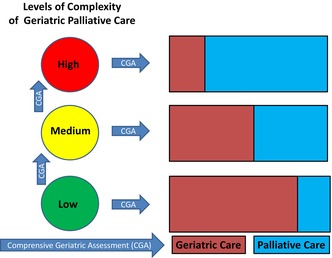

## 1 INTRODUCTION

We are currently experiencing significant demographic shifts that are leading to profound epidemiological changes. The progressive aging of the population has resulted in an increased prevalence of chronic degenerative diseases, leading to complex multipathological conditions.^1^ These demographic changes have already had a major impact on the development of palliative care, and this impact is expected to grow in the future.^2^, ^3^ A significant number of older persons spend the last days of their lives in hospitals and here often receive aggressive medical treatments.^4^ Projections to 2030 confirm that this trend will continue. How many of these people were residents of skilled nursing facilities before the admission to hospital immediately before their death is not clear. This also highlights the challenges faced in ensuring sufficient caregivers in the community, as the majority of these people will be older women living alone.^3^ This demographic shift is occurring in conjunction with global social, cultural, and economic changes.^5^ The process of aging is often accompanied by progressive frailty and development of multiple comorbidities, and many older persons live for extended periods with complex chronic illnesses..^6^, ^7^ It is crucial to recognize the different phases of disease progression (from chronicity to end‐of‐life care) in order to prevent further deterioration of quality of life or premature death. Recent years have seen a growing focus on geriatric and palliative care due to their potential to address the complex health care needs of aging populations, to provide interventions that manage comorbidities, alleviate symptoms, and enhance quality of life.^8^ Palliative care requires an interdisciplinary approach that prioritizes symptom management and quality of life for patients and their caregivers. The integration of geriatric and palliative care models can lead to improved outcomes for patients and increased satisfaction among patients and their families,^8^ and it is crucial for clinicians to actively involve elderly patients and their caregivers in the development of innovative palliative care interventions.^9^ Comprehensive Geriatric Assessment (CGA) guidelines can be a valuable tool for developing personalized care plans for older adults receiving palliative and supportive care. A more pragmatic perspective on this state of “near death” should focus on frailty rather than the exact timing of death. From this point of view, those who suffer from serious illnesses at the end of their lives can be identified not by a certain prediction of when death will occur, but by existing in a state of “living on thin ice”—enduring extended periods of ailment or disability, reduced functionality, and potential symptom exacerbation, any of which could prove fatal. This state could persist for several years or for few days. This means that patients in the terminal phase of a serious illness or of a prolonged disability may exhibit symptoms that are characteristic of the end‐of‐life stage for a wide range of time.^10^

This collaborative approach not only strengthens the evidence base but also promotes individualized care delivery strategies.^10^ Health status, quality of life, and care needs are strongly influenced by economic and social factors. It is important to assess the complexity of patient's needs through a comprehensive multidimensional evaluation and tailor the intensity of palliative care accordingly.^11^ Different care settings for older adults, such as hospitals, long‐term care facilities, and home care, have been viewed as interconnected systems that can benefit from “E‐Geriatrics” approaches. This involves telemedicine and artificial intelligence to improve the management of older patients and strengthen interdisciplinary collaboration.^12^, ^13^, ^14^

## 2 ITALIAN GUIDELINES ON CGA AND PALLIATIVE CARE

Recently, the Società Italiana Ospedale e Territorio (SIGOT) and the Società Italiana di Medicina Generale (SIMG) promoted a collaborative initiative to write the first guidelines on the CGA. Twenty‐five Italian scientific societies were involved, and experts from the Italian Institute of Health provided methodological support.^15^ These guidelines propose recommendations on the most appropriate multidimensional assessment tools for health care and social professional caring for older individuals in various settings. The review questions were formulated based on the identification of relevant PICOs (Patient or Population, Intervention, Comparison, and Outcome) specific to each setting. According to this methodological strategy, different panels of experts determined the outcomes of interest based on their clinical expertise. Structured searches were conducted on databases such as Cochrane Library, PubMed, and Embase using keywords related to geriatric assessment, and multidimensional geriatric assessment across nine clinical settings. In particular, Section 5 focused on patients in hospice and other palliative care facilities. The literature search identified 1337 randomized controlled and observational trials involving individuals aged 65 and older receiving hospice and palliative care. For Question 9: What is the usefulness of Comprehensive Geriatric Assessment in hospice patients? And in the network of other palliative care? The PICOs questions were: **P**: elderly patients admitted to hospice and other palliative care network; **I**: Comprehensive Geriatric Assessment; **C**: No Comprehensive Geriatric Assessment; **O**: Mortality (all causes and specific cause), functional status, quality of life, number of medications prescribed, appropriateness of prescribed medications, frequency of use of restraints (Table 1). None of the identified studies met the criteria for inclusion. These findings underscore the need for further research to determine the potential benefits of a multidimensional approach for patients in hospice and other palliative care settings.

**TABLE 1:** PICO Questions.

For Question 9: What is the usefulness of comprehensive geriatric assessment in hospice patients? And in the network of other palliative care?	
**P**	Elderly patients admitted to hospice and other palliative care network
**I**	Comprehensive Geriatric Assessment
**C**	No Comprehensive Geriatric Assessment
**O**	Mortality (all causes and specific cause), functional status, quality of life, number of medications prescribed, appropriateness of prescribed medications, frequency of use of restraints

## 3 DISCUSSION

Theoretical models are not suitable for Geriatric Palliative Care (GPC), given the level of care required for individuals living with life‐limiting diseases and progressive care needs. This care must be tailored to align with appropriate specialist involvement.

**FIGURE 1:**
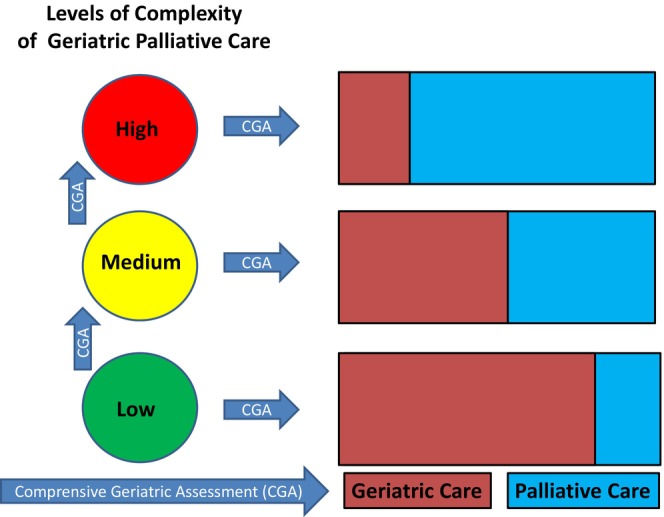
Model for levels of complexity of geriatric palliative care.

In Figure 1, we present a potential model of GPC structured into three different levels of care complexity (Low, Medium, and High), useful in depicting a hypothetical progression of geriatric/palliative care intensity.

The green sphere (beginning of the pathway) represents the majority of individuals receiving palliative care at an early stage of illness or with a relatively stable clinical condition. These patients require minimal specialist care and can effectively manage their symptoms at home and in the community with the support of primary health care teams and of caregivers. This majority of patients can benefit from a “palliative approach to care” that integrates essential aspects of palliative care into nonspecialist care, focusing on patient‐ and family‐centered care with an emphasis on quality of life. More complex care needs (yellow sphere) are primarily managed by nonspecialist Palliative Care doctors/teams. Moderately complex needs may require and necessitate the expertise of a specialist Palliative Care team. Lastly, red sphere refers to patients with complex needs requiring intensive, comprehensive care from a specialized Palliative Care team. General practitioner and geriatrician help to ensure continuity of care and support relational aspects.

Once a potential model is established, CGA is crucial in determining the optimal timing for care interventions.^13^, ^14^ In addition, the principles of GPC are to be integrated into research and health care planning to enhance the quality of services for a growing and different demographic with multiple health conditions. Ensuring access to care requires a focus on improving the organization and effectiveness of GPC services.

The everyday practice of palliative care proposes several complex challenges, and difficulties are present in understanding, describing, and treating them. The solutions are often not straightforward and can be hard to come by. Palliative care professionals play a crucial role in the development of integrated and cost‐effective models of GPC by adopting the principles of complexity science.^16^ The diagnosis of complexity in this context requires a thorough and interdisciplinary assessment to identify the various factors contributing to the patient's complex condition.^17^ Complexity science in the field of medicine offers valuable insights into the dynamic interplay between individual and collective needs, the complexities of decision‐making in the face of limited information, and the uncertainties surrounding therapeutic outcomes.^18^ As known, the CGA takes into account various multidimensional parameters such as functional status, multimorbidity, and socio‐economic factors, thus improving the prognostic stratification of elderly patients.^19^, ^20^ Early prognosis in older individuals is becoming increasingly important for guiding clinical decisions.^21^ The complexity present in palliative care is influenced by factors related to the patient and their family, as well as environmental considerations that encompass professional, health care, and community contexts.^22^

## 4 CONCLUSION

In the past, “Geriatrics” and “Palliative Care” traveled on parallel tracks. Geriatric culture has usually been attentive to progressive frailty, vulnerability, comorbidity, cognitive impairment, and polypharmacy risk. Palliative care has usually focused on quality of life and symptom management in patients with severe disease or limited life expectancy. The current public health context requires an open dialogue between Geriatrics and Palliative Care, challenging misconceptions and supporting holistic care. The holistic approach can transform how we address the end of life, ensuring that elderly and frailer individuals experience a transition that is not only medically managed but respectful of their spirituality, their emotions and in a word, of all their needs.

Consequently, GPC requires a complete assessment that includes various areas of patients’ needs, carried out through a CGA that uses validated tools. It is undergoing a necessary transformation in response to the evolution of the dynamics of phases of death, with increasingly difficult therapeutic decisions, punctuated by multiple psychosocial problems and the spiritual emptiness that characterizes our society. The culture of GPC care must be created and implemented before the clinical challenges become overwhelming. This is possible by spreading the art of goal management, and also based on the use of CGA. This means that patient management must be based not only on the chronological age but also on the correct multidimensional approach, which allows us to weigh the frailties better and choose the most appropriate path.

Health needs are not static but evolve to respond to patients’ needs. The demographic, social, and health frameworks will continue to evolve over time and need to be updated by health systems. This is also true for the GPC complexity, no doubt.

As the Italian guidelines highlighted, the road ahead is still very long. We hope that our reflections on the CGA use in GPC may be a starting point for an open and continuous dialogue between all parties involved.

## AUTHOR CONTRIBUTIONS

Conceptualization, AC, CM, and GR; writing—original draft preparation, AC, VM, CM, CR; writing—review and editing, AC, VM, CM, CR; supervision, AC, CM, GR. All authors have read and agreed to the published version of the manuscript.

## FUNDING INFORMATION

This research received no external funding.

## CONFLICT OF INTEREST STATEMENT

The authors declare no conflicts of interest.

## SPONSOR'S ROLE

No funders had a role in writing the letter or the decision to submit it for publication.

## Data Availability

No data has been used for this perspective.
